# Cancer@Work — a nurse-led, stepped-care, e-health intervention to enhance the return to work of patients with cancer: study protocol for a randomized controlled trial

**DOI:** 10.1186/s13063-016-1578-8

**Published:** 2016-09-15

**Authors:** Sietske J. Tamminga, Jan L. Hoving, Monique H. W. Frings-Dresen, Angela G. E. M. de Boer

**Affiliations:** Coronel Institute of Occupational Health, Academic Medical Center, University of Amsterdam, Amsterdam, The Netherlands

**Keywords:** Cancer survivors, Employment, Return to work, E-health, Psycho-oncological care, Study protocol, Randomised controlled trial

## Abstract

**Background:**

Although the importance of work for patients with cancer is nowadays more acknowledged both in the literature as well as in cancer survivorship care, effective interventions targeting the return to work of these patients are still scarce. Therefore, we developed a nurse-led, stepped-care, e-health intervention aimed at enhancing the return to work of patients with cancer. The objective of this study is to describe the content of the intervention and the study design used to evaluate the feasibility and (cost) effectiveness of the intervention.

**Methods:**

We designed a multi-centre randomised controlled trial with a follow-up of 12 months. Patients who have paid employment at the time of diagnosis, are on sick leave and are between 18–62 years old will be eligible to participate. After patients have signed the informed consent form and filled in the baseline questionnaire, they are randomly allocated to either the nurse-led, stepped-care, e-health intervention called Cancer@Work, or care as usual. The primary outcome is sustainable return to work. Secondary outcomes are sick leave days, work ability, work functioning, quality of life, quality of working life and time from initial sick leave to full return to work without extensive need for recovery. The feasibility of the Cancer@Work intervention and direct and indirect costs will be determined. Outcomes will be assessed by questionnaires at 3, 6, 9 and 12 months of follow-up.

**Discussion:**

The results of this study will provide new insights into the feasibility and (cost) effectiveness of Cancer@Work, a nurse-led, stepped-care, e-health intervention for cancer patients aimed at enhancing their return to work. If proven effective, the intention is to implement the Cancer@Work intervention in usual psycho-oncological care.

**Trial registration:**

NTR (Netherlands Trial Registry): NTR5190. Registered on 18 June 2015.

## Background

In the last decades, the survival rates of cancer have improved significantly for most cancer types [1]. For that reason, a growing number of cancer survivors of working age are now able to remain in or return to work. However, a considerable number of patients with cancer still experience difficulties getting back to work [2] or they become unemployed [3] due to, for instance, cancer-related side effects or an insensitive work environment [4]. It is important to diminish these adverse work outcomes for patients with cancer, as work is both a key aspect of cancer survivorship and is also associated with higher levels of quality of life in these patients [5].

Although the importance of work for patients with cancer is nowadays more acknowledged both in the literature as well as in cancer survivorship care, interventions that are primarily aimed at enhancing return to work of cancer patients are still scarce [6]. In the past few years, a few studies reported results of an intervention primarily aimed at enhancing the return to work of cancer patients [6–8]. These studies reveal that it is feasible to deliver a work support intervention during cancer care that is highly appreciated by patients [7, 9]. However, these work support interventions need improvement, as most did not show effectiveness on return to work [9].

Most work support interventions for patients with cancer have been delivered to all patients in the same manner [6, 10]. Nevertheless, it is known that patients widely differ both in the number and type of work-related problems they experience upon their return to work [11] as well as in the duration from initial sick leave to sustainable return to work [12]. These differences indicate that a stepped-care intervention, in which the intensity of the intervention is tailored to the patients’ needs, might be more effective and efficient. Such a tailored intervention may lead to more effective return-to-work outcomes.

Previous studies on cancer and work have taught us three main things. First, self-assessed work ability is an important prognostic factor for return to work, irrespective of clinical characteristics [7, 13]. This finding has led to the hypothesis that return to work is influenced by patients’ expectations and beliefs regarding return to work and that addressing misconception or improving someone’s self-efficacy would enhance return to work [13]. A successful approach to increase someone’s self-efficacy is the involvement of self-management interventions [14] which might be based on cognitive behavioural techniques [15] or problem-solving techniques [15, 16]. Second, both employers and colleagues as well as cancer care providers are important stakeholders in the return to work of patients with cancer [11]. It has appeared difficult to connect with the workplace from the hospital [17], but a successful approach to solve this problem might be the use of integrative-care management [18]. Third, the longer the duration of sick leave, the more difficult it is to return to work [19]. Addressing return to work in an early phase during cancer treatment might therefore be the window of opportunity. Moreover, two studies showed that cancer patients appreciated receiving information on their return to work during early cancer treatment [8, 17]. For these reasons, we choose to develop an early intervention based on the theory of self-management and integrative-care management.

Numerous studies have demonstrated that e-health is a suitable way of delivering self-management interventions for various conditions, including cancer [20, 21], and to deal with various problems, including return to work [22, 23]. In addition, e-health proved suitable for delivering an integrative-care management intervention as well [24]. Based on these findings, we choose to deliver the intervention as an e-health intervention. We define e-health as the process of providing the intervention partly over the Internet including interaction with health care providers.

### Objective

The first objective of this paper is to describe the content of Cancer@Work, a nurse-led, stepped-care, e-health intervention aimed at enhancing the return to work of patients with cancer. The second objective is to present the study design aimed at evaluating the Cancer@Work intervention in terms of feasibility and (cost) effectiveness.

## Method

### Content of the Cancer@Work intervention

The Cancer@Work intervention has been developed based on a non-systematic review on the use of and compliance with e-health interventions, a non-systematic review on cancer and work and on semi-structured interviews with experts, cancer survivors, occupational physicians and supervisors. Development of the nurse-led, stepped-care, e-health intervention Cancer@Work has been described extensively elsewhere [25].

The Cancer@Work intervention is an e-health intervention blended with care from a specialised nurse of the treating hospital or another care provider (e.g. social worker), depending on the local setting. For readability we will refer to this care provider as a specialised nurse. The specialised nurse will (1) answer questions, (2) monitor and supervise use of the Cancer@Work intervention, (3) provide personal feedback on assignments of the Cancer@Work intervention and (4) encourage patients to comply with the intervention. To be able to blend their care with the Cancer@Work intervention, specialised nurses have access to a special section of the e-health intervention with which they are able to (1) see whether patients have used the Cancer@Work intervention, (2) see which functionalities each patient has used, (3) evaluate the content of some of the assignments, (4) answer questions from patients, (5) send messages to patients and (6) receive support from and answers to questions from an oncological occupational physician.

The intervention comprises two steps. Whether or not the intervention goal was met with step 1 is measured based on two indicators assessed by the specialised nurse: (1) no return to work and/or (2) problems experienced upon returning to work. If either indicator is affirmative, patients will enter step 2.

### Step 1: Information and advice in the Cancer@Work intervention and integrative-care management

The focus of step 1 is to deliver patient-tailored information on cancer and work with the help of the Cancer@Work intervention and to address misconceptions on cancer and work. Functionalities of the Cancer@Work intervention include assignments to create insight and to stimulate patients to take action on the following topics: (1) possible financial consequences of sick leave, (2) rights and obligations according to the Dutch social security system when sick-listed (tailored to the patient’s situation, i.e. a fixed versus temporary employment contract), (3) individual importance of work and (4) return-to-work plan. Furthermore, the Cancer@Work intervention includes a library with background information and a self-test to create awareness of the abilities to return to work and to restructure cognitions. The library includes, for instance, information on work adjustments, social support and legal and insurance issues, and it is conveniently arranged and has a search function. Additionally, patients can learn from former patients by means of advice, answers to frequently asked questions and the documentary ‘Irrevocable’ on experiences of cancer survivors upon returning to work. Patients are also able to keep a diary and to have a look at assignments that they have previously made. Finally, the Cancer@Work intervention offers patients the ability to send private messages to their specialised nurse. Following a patient’s first login, a welcome text and an instruction video explaining how to use the e-health intervention will be available.

Integrative-care management implicates the involvement of the patient’s supervisor, occupational physician and general physician. To accomplish this, the patient will be encouraged to invite his/her supervisor, occupational physician and/or general physician to use a public website, specially developed as part of the e-health intervention, containing information on cancer and work for supervisors, occupational physicians and general physicians (http://www.kanker-werk.nl/). When a patient invites his supervisor, occupational physician and/or general physician, they will receive an email with a short explanation of the aim of the Cancer@Work intervention. Once a patient has invited his supervisor, occupational physician or general physician, the Cancer@Work intervention offers the ability to send private messages to the stakeholder whom the patient has invited.

### Step 2: Problem solving in extended Cancer@Work intervention

The main functionality of the second step is a self-management assignment based on the problem-solving technique and blended care. Patients themselves actively work on their problem with the aid of the Cancer@Work intervention and the additional support of their specialised nurse. The ultimate goal is to create a strategy to manage the patient’s problems that inhibit a successful return to work. The self-management program is split into three assignments. First, patients formulate problems and opportunities regarding work. Second, patients formulate solutions for work-related problems. Third, patients generate a plan to execute the chosen solutions with regard to return to work. During this process their specialised nurse will provide personalised feedback after each assignment (online or face-to-face), monitor and supervise the progress and encourage patients to comply with the intervention.

#### Conditions of use

There is no prescribed use of the Cancer@Work intervention in terms of length, frequency or duration. Patients themselves decide when to use the Cancer@Work intervention and which content to use. They will go through the Cancer@Work intervention at their own pace and move from step 1 to step 2 if indicated. The Cancer@Work intervention will be available for 12 months.

#### Enhancing compliance

Every month a standardised email will be sent to patients as a reminder of the functionalities of the Cancer@Work intervention, if patients gave permission for these emails. These reminders intend to increase compliance with the intervention. Additionally, patients can ask their specialised nurse for personal coaching at any time during availability of the Cancer@Work intervention.

#### Privacy and support for the Cancer@Work intervention

For technical questions only, patients are able to contact the researcher by email. All patients are registered by the researcher and receive a unique username and password (changeable later). Patients are able to log in to the secured webpage by use of their unique username and password; then verification by text message will take place. The patient’s specialised nurse is able to view their profile and provide personalised feedback.

### Study design to evaluate the intervention Cancer@Work

For the description of the design of our evaluation study, we used the items of the CONSORT statement [26] as well as the SPIRIT checklist [27, 28] for improving the quality of reporting randomised trials.

### Organisation study

**Fig. 1:**
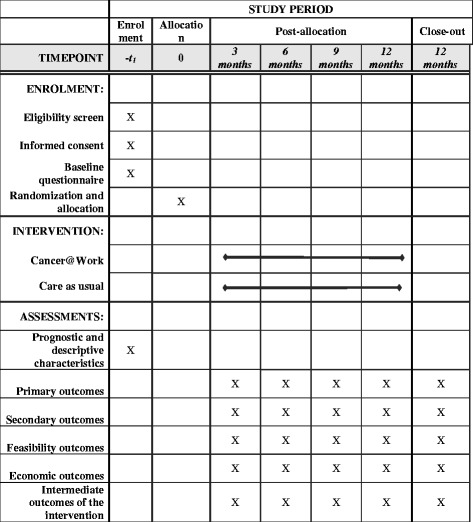
Schedule of enrolment, intervention and assessments

The design of this study is a non-blinded, multi-centre, randomised controlled trial with a follow-up of 12 months. An overview of consent, screening, enrolment, intervention, timing of measurements and data collection is shown in Fig. 1. The study will be conducted at the following hospitals from various geographical regions in the Netherlands: Academic Medical Center, Albert Schweitzer Hospital, Reinier de Graaf Groep, Flevoziekenhuis, the Antoni van Leeuwenhoek Hospital and Ter Gooi Hospital. The intervention group will receive the nurse-led, stepped-care, e-health intervention Cancer@Work, and the control group will receive care as usual, which is standard psycho-oncological care. The medical ethics committee of the Academic Medical Center approved the study and the conduction of the study at all participation hospitals. The board of management of the above-mentioned participating hospitals advised positively about local feasibility. Patients will sign an informed consent form before participating.

### Recruitment of study population

**Fig. 2:**
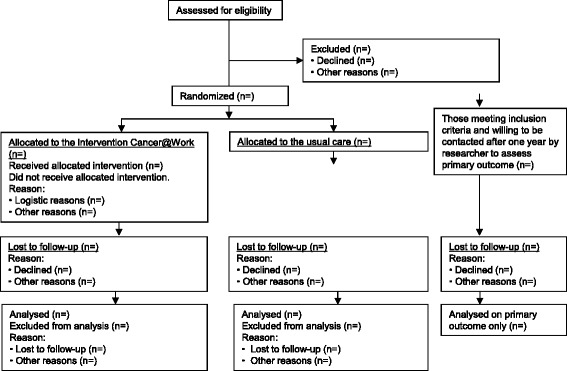
Participant flow diagram

The treating physician or nurse will check each patient’s eligibility at the outpatient clinic a few weeks after the first cancer treatment, which consists in most cases of surgery. The treating physician or nurse will inform all eligible patients about the study and will ask for consent to be contacted by the researcher. After telephonic contact by the researcher, the researcher will include patients in the study if they are willing to participate and after they have provided the signed informed consent form. Thereafter, the patient will be randomised to either the intervention group or to the control group. Patients who meet the eligibility criteria but are not willing to participate will be asked if they are willing to answer a few questions by telephone about their work situation at baseline and to answer by telephone a few questions about their work situation at 12 months of follow-up. We choose this strategy to be able to comprehend if a selective population of cancer patients have participated in our evaluation study. The participant flow diagram is shown in Fig. 2.

### Participants

Eligible for this study will be patients with a primary diagnosis of cancer who will be treated with curative intent at the hospital of recruitment, between 18–62 years old, in paid employment or self-employed and who have sufficient knowledge of the Dutch language. Patients will be excluded if they have a severe comorbidity or if they have no Internet access.

### Randomisation, blinding and treatment allocation

The researcher will perform randomisation by means of the computerised randomisation program Alea [29]. By using this computerised program, randomisation is definite and allocation concealment is not possible. To prevent unequal randomisation, randomisation will be stratified by hospital department and age (cut-off value 50 years), since cancer diagnosis and higher age are important prognostic factors for delayed return to work [30]. To equalise group sizes, blocks of 6 will be used. Patients, care providers and the researcher will not be blinded for group assignment.

### Sample size

On average, we will include cancer patients at 6 weeks or 42 days after diagnosis, and the follow-up time for the primary outcome return to work will be 12 months after inclusion, hence 12 months and 42 days after diagnosis. Earlier studies in our hospital showed that return to work for patients with various cancer types was 64 % at 12 months of follow-up from diagnosis [28]. In addition, earlier studies in our hospital showed that return to work in the first 42 days is around 8 % [28]. Therefore, the return to work for the control group was set at 64 % + 8 % = 72 %. In a study of Nieuwenhuijsen et al. [29], an intervention group of 35 radiotherapy patients received a supportive work-related intervention in a hospital. Time between diagnosis and the end of treatment was a median 42 days. Return to work was measured between the end of treatment and work resumption and was 89 % after 12 months. Therefore, the return to work for the intervention group in our intervention was also set at 89 %.

Based on our earlier studies on consecutive cancer patients, a power analysis indicated that we should include a total of 170 patients with a follow-up of 1 year after inclusion to indicate a difference of 89 % return to work in the intervention group versus a 72 % return-to-work rate in the control group, with a power of 80 % and *p* < 0.05. Accounting for 10 % loss to follow-up, 190 patients should be included.

### Contamination

Participants will be able to use any co-intervention they wish. Since it is likely that other (vocational) rehabilitation programs will have a significant effect on return to work, participation in one of these co-interventions will be monitored by means of follow-up questionnaires.

In theory, participants could provide knowledge about the intervention and even access to Cancer@Work to participants in the control group. However, as participants will be recruited individually via outpatient clinics of hospitals and not, for instance, via support groups, the chance that a patient from the intervention group will know a patient from the control group is nil. Nonetheless, we will ask participants in the control arm, via follow-up questionnaires, whether they received any support related to return to work.

### Usual care in the Netherlands

Currently work-related issues are not structurally addressed as part of cancer (survivorship) care in the Netherlands. However, it is becoming more common to discuss this issue with a care provider at the hospital or to receive support by a reintegration office specialised in patients with cancer.

All sick-listed employees should have an occupational physician who should legally advise them about return to work according to the blueprint of evidence-based guidelines of the Dutch Association of Occupational Physicians [31]. The Improved Gatekeepers Act covers sick leave in the Netherlands and is in force during the first 2 years of sick leave. The act states that a sick-listed employee cannot be discharged and receives at least 70 % of his/her wage. The Improved Gatekeepers Act states that employers and sick-listed employees are together responsible for work resumption, which means that both parties can be sanctioned.

In the Netherlands, the social security system also provides a safety net for sick-listed workers without an employment contract according to the Improved Gatekeepers Act. Application of the Act is the same, except that these workers do not have an employer/workplace to return to. Sick-listed workers without an employment contract receive 70 % of their last daily wage during the first 2 years of sick leave. The Dutch Social Security Agency pays for the sick leave, is responsible for executing obligatory occupational health actions and should facilitate work resumption.

Self-employed persons can be privately insured against sickness absence, but most are uninsured because of the high costs. If a person is privately insured, it depends on the insurance policy as to when someone receives a supportive income, how much this supportive income is and whether or not he/she receives sickness absence counselling and vocational rehabilitation guidance.

### Training specialised nurses to carry out the Cancer@Work intervention

Specialised nurses will be educated on the work-related issues of cancer patients, the social security system of the Netherlands, how to coach patients with self-management based on problem-solving techniques, how to deliver personalised feedback and how to encourage patients to comply with the Cancer@Work intervention [32]. Training of the specialised nurses will consist of two half-day training courses, supervised by an experienced trainer and a member of the research team.

### Assessment of the outcomes

Data will be both self-reported and collected by recording use of the Cancer@Work intervention by participants and recording the content of the assignments made by participants. The self-reported outcomes will be assessed at baseline and at 3, 6, 9 and 12 months of follow-up using questionnaires that will be completed by participants randomised to the intervention group and control group with online software. All participants will receive an email with a link to complete the questionnaire online if they indicate to prefer questionnaires online; otherwise, they will receive paper questionnaires. Completing a questionnaire will take approximately 30 minutes.

#### Feasibility evaluation

The following feasibility outcomes will be measured according to the Bowen scheme [33]: acceptability, demand, practicality and compliance with the Cancer@Work intervention. By acceptability we mean general usefulness of the Cancer@Work intervention and of each functionality, general user-friendliness of the Cancer@Work intervention and of each functionality, appropriateness of the intervention for the patient, satisfaction with support of their specialised nurse in their use of the Cancer@Work intervention, information that should be useful to add to the Cancer@Work intervention, functionalities that should be useful to add to the Cancer@Work intervention and general ideas to improve the Cancer@Work intervention. All these parameters will be measured at the patient level with self-reported questionnaires at follow-up.

We define demand as the extent to which the Cancer@Work intervention has actually been used and include number of log-ins into the Cancer@Work intervention, use of each functionality of the Cancer@Work intervention (number) and the number and the content of assignments. These will all be measured based on a website monitoring program. To assess practicality we will evaluate the self-perceived knowledge and skills of the specialised nurse regarding the intervention, protocol for the specialised nurse, website for the specialised nurse and appropriateness of the training and preparation of the specialised nurse. These will all be assessed as part of the semi-structured interview with the specialised nurse at the end of the study.

Compliance by patients will be measured with a website monitoring program. Reasons for non-compliance will be measured based on self-reported questionnaires at follow-up as well as on whether or not use of the Cancer@Work intervention fits in the patient’s daily routine, since that is often a reason for not complying.

#### Effect evaluation

The primary outcome of sustainable return to work is measured as any return to work (yes/no) at follow-up independent to the number of contract hours which is sustained for at least 4 consecutive weeks without recurrent sick leave. Return to work is either partial return to work (working a part of contract hours) or full return to work (working all contract hours).

The secondary outcomes are full return to work (yes/no) at follow-up defined as working the hours stated in the work contract, time to any (either partial or full) return to work in days from the first day of sick leave to the date of any return to work and time to return to work in days from the first day of sick leave to the date of full return to work. These return-to-work measures must be sustained for at least 4 consecutive weeks. Furthermore, secondary outcome measures are work ability using the first three questions of the Work Ability Index (WAI) [34], work functioning using the Work Limitations Questionnaire (WLQ) [35, 36], quality of life using the SF-12 [37], quality of work life using the QWLQ-CS (De Jong et al. in preparation) [38] and the time from initial sick leave to full return to work without extensive need for recovery using a subscale of the ‘perception and assessment of work questionnaire’ (VBBA) [39].

#### Prognostic and descriptive characteristics

Prognostic characteristics are age, gender, marital status (married, single, widowed, divorced), bread winner status (yes, no, shared), education (7 categories), cancer diagnosis, treatment type (surgery, radiotherapy, chemotherapy, hormonal therapy, other), time since diagnosis (in months), treatment duration and comorbidity (13 diseases). Work-related factors include characteristics of the job (working hours per week, job position, company size, shift work (no, yes with/without night shifts), type of contract (permanent, temporary, self-employed), years in present position, years of work experience, and work adjustments), work demands (VBBA) [39], importance of work (0 (not important)–100 (most important)), current problems with work (yes/no), need for information or support regarding work-related problems (1 (no)–5 (very much)) and expectations regarding return to work in next 4 weeks (1 (definitely yes)–4 (probably not)). Fatigue is measured with the 20-item Multidimensional Fatigue Inventory (MFI) [40] with higher scores indicating more fatigue, depression is measured with the 20-item CES-D [41] with higher scores indicating more depressive symptoms and cognitive functioning at work is measured with the 20-item Cognitive Symptom Checklist-Work questionnaire [42]. The following descriptive characteristics will be measured: income (in euros per month) and work participation status at follow-up (employed, unemployed, disability pension, retired).

#### Economic evaluation

The economic evaluation will be conducted from a societal perspective. The following direct costs will be measured for both groups: costs to deliver the Cancer@Work intervention or costs to deliver care as usual and the following indirect costs: absenteeism, lost work productivity, lost earnings and work adjustments. The direct costs will be determined by means of the costs to deliver the Cancer@Work intervention (i.e. costs to develop and maintain the e-health intervention, average specialised nurse wage, the amount of time spent on each participant and training costs) or costs to deliver care as usual (i.e. average specialised nurse wage and the amount of time spent on addressing return to work of each participant). Absenteeism costs will be determined by means of total days on sick leave from the first day of sick leave until follow-up and on income and work productivity costs by the WLQ and on income [35, 36]. Additionally, lost earnings will be determined on the basis of the differences between income at baseline and income at follow-up. Work adjustments will be assessed by means of the cost of each work adjustment.

Direct costs will be acquired by the specialised nurses keeping track of the time spent on each participant and by the research team on keeping track of the costs to develop and maintain the Cancer@Work intervention and training costs to train specialised nurses to deliver the Cancer@Work intervention. Indirect costs will be acquired by a self-reported questionnaire completed by participants during follow-up.

#### Intermediate effect of the Cancer@Work intervention on self-management and work-related self-efficacy

The Cancer@Work intervention is a self-management intervention based on problem-solving techniques and cognitive behavioural techniques. The key mechanisms of the Cancer@Work intervention are therefore self-management skills and work-related self-efficacy. To measure this change in self-management skills, we will measure perceived confidence in own self-management skills on a visual analogue scale of 0 (no confidence) to 100 (high confidence). Work-related self-efficacy will be measured with the 11-item questionnaire ‘expectations regarding work functioning’ [43] with higher scores indicating more self-efficacy regarding work.

### Statistical analysis

All analyses will be based on the intention-to-treat (ITT) principle. All baseline data and data regarding primary and secondary outcomes as well as data from the feasibility evaluation will be presented using descriptive statistics. Differences in baseline data between the intervention and control group will be assessed using Student’s *t* test for continuous data and the chi-squared test for categorical data.

#### Feasibility evaluation

The following feasibility outcomes will be described according to the Bowen scheme [33]: acceptability, demand, practicality and compliance with the Cancer@Work intervention. The following baseline parameters will be analysed to verify whether they are related to non-compliance: treatment type, treatment duration, age, education, gender, need for cognition regarding return to work, need for support with return to work, existence of problems with work, expectations concerning return to work, expectations concerning the Cancer@Work intervention, Internet skills and self-management skills. The following parameters measured at follow-up on patient level by means of self-reported questionnaires will be related to non-compliance: whether or not their expectations about the Cancer@Work intervention are fulfilled, work situation at follow-up and satisfaction with the Cancer@Work intervention.

#### Effectiveness

The relative risk and 95 % confidence interval for returning to work at 12 months of follow-up will be calculated for the intervention group versus the control group. In addition to this ITT analysis aimed at ’all-cause return to work’, the analysis will be performed excluding those patients who have died during follow-up and those who have a life expectancy of less than a few months, because they will not return to work.

To assess whether the Cancer@Work intervention is more effective for those who need it most, a planned subgroup analysis for patients with a high risk of not returning to work will be performed. This subgroup will be identified based on having physical heavy work, low work ability at baseline, high levels of fatigue, depression and cognitive problems at work at 3 and 6 months of follow-up and a long duration of cancer treatment [13, 30]. In addition, to ascertain whether the Cancer@Work intervention is more effective for various subgroups of patients who are more or less prone to benefit from self-management, a subgroup analysis will be done for young versus old, male versus female and lower education level versus higher education level [44].

#### Effectiveness secondary parameters

The number of days until participants’ return to work (either full or partial) assessed at 12 months will be analysed using the Kaplan-Meier survival method, and differences between groups will be tested with the log-rank test. Patients who dropped out of the study will be censored. If statistically significant differences in prognostic characteristics between the intervention and the control group are found at baseline, a Cox regression analysis will be performed including those prognostic parameters.

A longitudinal multilevel analysis will be used to examine differences between intervention and the control group with regard to improvement of work ability, work limitations, quality of work life and quality of life.

A cut-off score of ≥6 on the subscale need for recovery of the VBBA will be used to divide the group of participants who returned to work without extensive need for recovery versus the group of participants who returned to work with an extensive need for recovery plus the participants who did not return to work. The number of days until participants’ return to work without extensive need for recovery at 12 months will be analysed using the Kaplan-Meier survival method, and differences between groups will be tested with the log-rank test. If statistically significant differences in prognostic characteristics between the intervention and the control group are found at baseline, a Cox regression analysis will be performed including those prognostic parameters.

#### Economic evaluation

Direct and indirect costs will be summed for each participant. Using bootstrapping, mean differences in direct, indirect and total costs will be calculated between the control group and the intervention group [45]. Incremental cost-effectiveness ratios will be calculated by assessing the ratio between the differences in costs between the intervention and control group to the differences in return-to-work rates between the groups.

#### Intermediate effect of the Cancer@Work intervention on self-management skills and work-related self-efficacy

A longitudinal multilevel analysis will be used to examine differences between the intervention and the control group on self-management skills and work-related self-efficacy.

## Discussion

The objective of this study is to describe the content of the nurse-led, stepped-care, e-health intervention Cancer@Work and to explain the study design used to evaluate the feasibility and (cost) effectiveness of this intervention.

As far as we are aware, this is the first e-health intervention especially developed for patients with cancer aimed at enhancing their return to work. Furthermore, a stepped-care intervention for supporting return to work is innovative as well. The Cancer@Work intervention provides an easily accessible intervention to deal at an early stage with work-related problems that might arise. When it is evident that return to work is hampered, this stepped-care intervention provides more intense problem-solving support. Additionally, the Cancer@Work intervention might bridge the gap between primary care and occupational health care, which is important, as both stakeholders are important for a return to work. The results of this study will provide new insights into the feasibility and effectiveness of a stepped-care approach and an e-health intervention for cancer patients aimed at enhancing their return to work.

Compliance is one of the main difficulties of an e-health intervention. Duffecy et al. tackled this problem successfully by means of supportive accountability [46]. This method involves a group start of the e-health intervention; patients could buzz each other when they did not log in for a certain amount of time. Unfortunately, our e-health intervention is not suitable for supportive accountability, as it cannot be used with a group start; return-to-work trajectories differ widely among patients, and our e-health intervention is not a fixed program. However, we tried to minimize the risk of low compliance by combining the e-health intervention with face-to-face contact, so-called blended care, by sending reminders to patients, by tailoring information to the patient (i.e. fixed versus temporary employment contract) and by involving all end users in the development of the intervention [47].

Internet illiteracy is associated with a lower education level, while research indicates that self-management interventions might be more effective for patients with a lower educational level [44]. For that reason, we have spent extra time and effort in the development of the Cancer@Work intervention component to ensure that it was very easy to use. Moreover, one of the functionalities of the Cancer@Work intervention component is the ability for patients to contact their specialised nurse or the researcher at any time during weekdays when they have problems with the content or technical problems.

The strengths of our study design are the inclusion of patients from various hospitals from various geographical regions in the Netherlands, implementing the Cancer@Work intervention into usual care without much deviation from usual care and the follow-up of patients unwilling to participate in the trial but willing to fill in baseline and follow-up questionnaires.

A limitation of our study design is the inability of blinding patients and specialised nurses to the outcome of the randomisation. This might cause contamination between groups. Alternatives of randomisation on a patient level are cluster randomisation or a step-wedge design. A difficulty of cluster randomisation is the need for comparable clusters [48], which is not possible for this study on the hospital level as patient characteristics differ between hospitals. Likewise, a difficulty of a step-wedge design is the need for a larger sample size, starting with all clusters (i.e. hospitals) at the same time and an equal distribution of patients between clusters, which is burdening and results in complex statistical analyses [49]. For these reasons we believe that randomisation on the patient level is the best option for this study. Moreover, although specialised nurses might alter their usual care due to participation in the study, patients allocated to the control group do not have access to the Cancer@Work intervention.

If proven effective, the Cancer@Work intervention will be implemented in usual care. The e-health intervention might also be incorporated as a work-module into broader psychosocial e-health interventions for patients with cancer.

## Trial status

The study is currently recruiting patients. Inclusion started on 1 September 2015.

## Abbreviations

CES-D: Center for Epidemiological Studies-Depression Scale
CONSORT: Consolidated Standards of Reporting Trials
ITT: Intention to treat
QWLQ-CS: Quality of Working Life Questionnaire for Cancer Survivors
SF-12: Short form 12
SPIRIT: Standard Protocol Items: Recommendations for Interventional Trials
VBBA: Perception and assessment of work questionnaire (in Dutch: Vragenlijst Beleving en Beoordeling van de Arbeid)
MFI: Multidimensional Fatigue Inventory
WAI: Work Ability Index
WLQ: Work Limitations Questionnaire
